# Disease Activity-Associated Alteration of mRNA m^5^ C Methylation in CD4^+^ T Cells of Systemic Lupus Erythematosus

**DOI:** 10.3389/fcell.2020.00430

**Published:** 2020-06-05

**Authors:** Gangqiang Guo, Huijing Wang, Xinyu Shi, Lele Ye, Kejing Yan, Zhiyuan Chen, Huidi Zhang, Zibing Jin, Xiangyang Xue

**Affiliations:** ^1^School of Life Sciences and Technology, Tongji University, Shanghai, China; ^2^Department of Microbiology and Immunology, Institute of Molecular Virology and Immunology, Institute of Tropical Medicine, School of Basic Medical Sciences, Wenzhou Medical University, Wenzhou, China; ^3^Kidney Disease Center, The First Affiliated Hospital, College of Medicine, Zhejiang University, Hangzhou, China; ^4^Department of Gynecologic Oncology, Wenzhou Central Hospital, Wenzhou, China; ^5^Department of Nephrology, The First Affiliated Hospital, Wenzhou Medical University, Wenzhou, China; ^6^Laboratory for Stem Cell and Retinal Regeneration, Institute of Stem Cell Research, Division of Ophthalmic Genetics, The Eye Hospital, Wenzhou Medical University, Wenzhou, China

**Keywords:** systemic lupus erythematosus, CD4^+^ T cell, epigenetics, 5-methylcytosine (m^5^C), NSUN2

## Abstract

Epigenetic processes including RNA methylation, post-translational modifications, and non-coding RNA expression have been associated with the heritable risks of systemic lupus erythematosus (SLE). In this study, we aimed to explore the dysregulated expression of 5-methylcytosine (m^5^C) in CD4^+^ T cells from patients with SLE and the potential function of affected mRNAs in SLE pathogenesis. mRNA methylation profiles were ascertained through chromatography-coupled triple quadrupole mass spectrometry in CD4^+^ T cells from two pools of patients with SLE exhibiting stable activity, two pools with moderate-to-major activity, and two pools of healthy controls (HCs). Simultaneously, mRNA methylation profiles and expression profiling were performed using RNA-Bis-Seq and RNA-Seq, respectively. Integrated mRNA methylation and mRNA expression bioinformatics analysis was comprehensively performed. mRNA methyltransferase NSUN2 expression was validated in CD4^+^ T cells from 27 patients with SLE and 28 HCs using real-time polymerase chain reaction and western blot analyses. Hypomethylated-mRNA profiles of NSUN2-knockdown HeLa cells and of CD4^+^ T cells of patients with SLE were jointly analyzed using bioinformatics. Eleven methylation modifications (including elevated Am, 3′OMeA, m^1^A, and m^6^A and decreased Ψ, m^3^C, m^1^G, m^5^U, and t^6^A levels) were detected in CD4^+^ T cells of patients with SLE. Additionally, decreased m^5^C levels, albeit increased number of m^5^C-containing mRNAs, were observed in CD4^+^ T cells of patients with SLE compared with that in CD4^+^ T cells of HCs. m^5^C site distribution in mRNA transcripts was highly conserved and enriched in mRNA translation initiation sites. In particular, hypermethylated m^5^C or/and significantly up-regulated genes in SLE were significantly involved in immune-related and inflammatory pathways, including immune system, cytokine signaling pathway, and interferon signaling. Compared to that in HCs, NSUN2 expression was significantly lower in SLE CD4^+^ T cells. Notably, hypomethylated m^5^C genes in SLE and in NSUN2-knockdown HeLa cells revealed linkage between eukaryotic translation elongation and termination, and mRNA metabolism. Our study identified novel aberrant m^5^C mRNAs relevant to critical immune pathways in CD4^+^ T cells from patients with SLE. These data provide valuable perspectives for future studies of the multifunctionality and post-transcriptional significance of mRNA m^5^C modification in SLE.

## Introduction

Accumulating evidence has revealed that post-transcriptional RNA modifications in mRNA may serve as novel gene expression regulators. Such epigenetic markers include N6-methyladenosine (m^6^A), N1-methyladenosine (m^1^A), 5-hydroxymethylcytosine (hm^5^C), 5-methylcytosine (m^5^C), pseudouridine (Ψ), and inosine (Roundtree et al., 2017). Specifically, as a novel modified form of cytosine, m^5^C methylation of mRNAs in eukaryotes mainly depends on catalysis via the RNA methyltransferase NSUN2 (known as an m^5^C “writer”) (Abbasi-Moheb et al., 2012; Auxilien et al., 2012; Flores et al., 2017). Moreover, m^5^C epigenetic modification plays a significant role in eukaryotic biological functions such as nuclear export regulation and protein transcription modulation (Squires et al., 2012; Flores et al., 2017; Trixl and Lusser, 2019). Additionally, several studies have demonstrated that mRNA modification by the epitranscriptome marker m^5^C constituted an integral part of gene regulatory networks in both mammals and plants (Amort et al., 2017; Cui et al., 2017; Dominissini and Rechavi, 2017).

Systemic lupus erythematosus (SLE) is an autoimmune disease with complicated clinical manifestations in which disease activity plays a key role in therapy and prognosis (Morel, 2017). Loss of the normal balance of activity in CD4^+^ T cells is associated with the development of SLE (Yin et al., 2015). In addition, epigenetics-based genetic predispositions and their interaction in T cells are thought to contribute to SLE pathogenesis and development (Lei et al., 2009; Yeung et al., 2019); these epigenetic modulators include DNA methylation, DNA 5-hmC, and altered long non-coding RNA expression (Coit et al., 2015; Zhao et al., 2016; Zhang et al., 2018; Guo et al., 2019; Ye et al., 2019). For example, the transcription factor RFX1 affects the epigenetic status of CD4^+^ T cells, which result in autoimmune responses in SLE (Zhao et al., 2010).

Compared to those in healthy controls (HCs) group, 5-hmC levels in genomic DNA of CD4^+^ T cells from patients with SLE present different profiles, which results in the aberrant regulation of gene transcription in SLE pathogenesis (Zhao et al., 2016). Similarly, differentially expressed DNA methylation in CD4^+^ T cells of patients with SLE contributes to the clinical phenotypes of this disorder and may reflect the potential mechanism (Zhao et al., 2014). Accordingly, epigenetic modification profiling in SLE has proven beneficial toward obtaining a better understanding of patients with different disease activity (Lawrence et al., 2016; Meroni and Penatti, 2016). However, few meaningful results regarding the association between CD4^+^ T cells of SLE and m^5^C modifications in mRNA have been reported.

In this study, we utilized mass spectrometry to identify that m^5^C mRNA methylation in SLE CD4^+^ T cells serves as a crucial form of epigenetic modification. Next, we analyzed m^5^C methylation distribution and variation in mRNA by bisulfite sequencing in CD4^+^ T cells from patients with SLE presenting different disease activity compared to those in HCs. Our data demonstrated significant differences of m^5^C patterns according to disease activity and that the affected mRNAs are involved in disease-associated pathways. Furthermore, our data also provided a novel viewpoint for identifying key m^5^C-marked genes that may participate in the pathogenesis of SLE.

## Materials and Methods

### Subjects and Information

For this study, we recruited 47 subjects with SLE, as detailed in Supplementary Table S1, from The First Affiliated Hospital of Wenzhou Medical University between December 2019 and February 2020. All patients fulfilled the diagnostic criteria of the American College of Rheumatology (Petri et al., 2012). In addition, disease activity was assessed according to the systemic lupus erythematosus disease activity index (SLEDAI) (Uribe et al., 2004) at the time of blood collection prior to administering glucocorticoids and immunosuppressive agents to the patients. A total of 46 age- and sex-matched HCs without heart failure, renal failure, arthralgia, or rheumatic disease, and free from inflammatory conditions were also recruited from same hospital. For mRNA liquid chromatography-coupled triple quadrupole tandem mass spectrometry (LC-MS/MS), RNA sequencing (RNA-Seq), and RNA bisulfite sequencing (RNA Bis-Seq) analyses, 20 patients with SLE and 18 HCs were divided into six groups [among these, patients with SLE were grouped according to their SLEDAI scores: patients with SLEDAI score < 5 were divided into SLE stable (SA) groups (SA1, SA2) and those with SLEDAI score ≥ 9 were allocated to SLE moderate/major active (SM-MA) groups (SM-MA1, SM-MA2)]. For each group, five samples of the same SLEDAI score were mixed and sequenced; similarly, each control group comprised nine mixed and sequenced samples of the age- and sex-matched HCs. In the study of the effect of NSUN2 expression levels on RNA aspects, 22 patients with SLE [10 inactive (SLEDAI score < 5) and 12 active (SLEDAI score > 5)] and 23 HCs were used for quantitative reverse-transcription polymerase chain reaction (qRT-PCR) detection. For evaluating the effects of NSUN2 expression levels on protein aspects, 5 patients with SLE [1 inactive and 4 active] and 5 HCs were used for western blot detection. This study was approved by the Medical Ethical Committees of The First Affiliated Hospital of Wenzhou Medical University. All participants in this research provided written informed consent.

### CD4^+^ T Cells and RNA Isolation

Peripheral blood mononuclear cells were isolated from HCs and patients with SLE using human peripheral blood lymphocyte separation medium (Tianjin Hao Yang Biological Manufacture, Tianjin, China) within 6 h of sample collection. Peripheral blood CD4^+^ T cells were then purified following immunomagnetic separation using a human CD4^+^ T cell isolation kit (BD Biosciences, San Jose, CA, United States) according to the manufacturer’s instructions. The isolated human primary CD4^+^ T cells were lysed in TRIzol reagent (Invitrogen Life Technologies, Grand Island, NY, United States) for RNA purification. The isolated RNAs were digested by DNase I (Invitrogen, Waltham, MA, United States) to remove residual DNA, then collected in 25 μL of DNase/RNase-free water. Isolated RNA was stored at −80°C for use.

### Analysis of mRNA Modifications via LC-MS/MS

Total RNA was extracted from each sample using TRIzol reagent/RNeasy Mini Kit (Qiagen, Hilden, Germany). Total RNA was quantified and qualified using an Agilent 2100 Bioanalyzer (Agilent Technologies, Palo Alto, CA, United States), NanoDrop ND-1000 (Thermo Fisher Scientific Inc., Waltham, MA, United States), and 1% agarose gel. mRNA was isolated from total RNA using the NEBNext Poly(A) mRNA Magnetic Isolation Module (E7490; NEB, Ipswich, MA, United States) according to the manufacturer’s protocol. Purified mRNA was hydrolyzed to single nucleosides which were then dephosphorylated by enzyme treatment. Pretreated nucleosides solution was deproteinized using a Sartorius 10,000 Da molecular weight cut-off spin filter (Göttingen, Germany). Analysis of nucleoside mixtures was performed using an Agilent 6460 QQQ MS with ESI Jetstream ionization operated in positive ion mode, with SB-Aq 3.5 μm 2.1 × 150 mm high-performance LC column (Agilent Technologies) conditions according to the following solvent gradient [Solution A, high-performance LC-grade water with the relevant amount of formic acid to obtain a final formic acid concentration of 0.1% (vol/vol); Solution B, 100% acetonitrile with the relevant amount of formic acid to achieve a final formic acid concentration of 0.1% (vol/vol)]. The voltages and source gas parameters were as follows: gas temperature, 350°C; gas flow, 7 l min^–1^; nebulizer, 40 psi; sheath gas temperature, 350°C; sheath gas flow, 11 l min^–1^; capillary voltage, 3,500 V; and VCharging, 500 V. The molecular transition ions were quantified in multiple reaction monitoring mode (Supplementary Table S2). Multiple reaction monitoring peak information of modified nucleosides for each sample was extracted using Agilent Qualitative Analysis software. Peaks with signal-to-noise ratio ≥ 5 were considered as detectable nucleosides. Peak areas were then normalized to the quantity of purified mRNA of each sample.

### Next Generation Sequencing for mRNA Gene Expression Profiling

Total RNA of each sample was extracted using TRIzol reagent/RNeasy Mini Kit/other kits. Total RNA (1 μg) with RNA integrity value > 6.5 was used for subsequent library preparation. Next generation sequencing library preparations were constructed according to the manufacturer’s protocol (NEBNext Ultra Directional RNA Library Prep Kit for Illumina). Differentially expressed transcripts were defined with a foldchange ≥ 1.2, and FDR ≤ 0.05, similar to previous studies (Chen X. et al., 2019). The detailed experimental procedure and analytic strategy can be found in our previous study (Guo et al., 2018).

### Sequencing of Bisulfite-Converted RNAs (RNA-Bis-Seq) and Bioinformatics Analyses

Briefly, mRNA was purified from total RNA using the Dynabeads^®^ mRNA Purification Kit (Thermo Fisher Scientific Inc.). mRNA was bisulfite-converted and purified using the EZ RNA Methylation Kit (Zymo Research, Los Angeles, CA, United States). RNA libraries were then constructed using the NEBNext^®^ Ultra II Directional RNA Library Prep Kit (NEB) according to the manufacturer’s instructions. The library quality was evaluated using the BioAnalyzer 2100 system (Agilent Technologies, Inc.). Library sequencing was performed on an Illumina HiSeq instrument with 150 bp paired-end reads. Paired-end reads were harvested from the Illumina HiSeq sequencer and quality controlled based on a Q score of Q30, followed by 3′ adaptor-trimming and removal of poor-quality reads using cutadapt software (v1.9.3). Next, clean reads of busulfite-treated libraries were aligned to the reference genome (UCSC HG19) using meRanGs (a component of meRanTK) software with default parameters. The methylation status of each C within the genome was extracted using meRanCall (a component of meRankTK) software. meRanCompare (a component of meRanTK) software was used to identify differentially methylated sites (DMSs). The Ensembl genome features were used to annotate the methylated sites and DMSs. Only sites with coverage depth (methylated C number + non-methylated C number) ≥ 10, m^5^C methylation level ≥ 0.1, and methylated cytosine depth ≥ 5 were considered as credible m^5^C sites. Each group contained two sample replicates, and only overlapping m^5^C sites between two sample replicates were used to the following analyses. The m^5^C sites were annotated by applying BEDTools intersectBed (Quinlan and Hall, 2010). The distribution of m^5^C sites was analyzed as previously described (Yang et al., 2017). To acquire the sequence preference proximal to m^5^C sites, 21 nt sequences centered on each m^5^C site were extracted using BEDTools (Quinlan and Hall, 2010); and logo plots were generated using WebLogo^1^ and motif enrichment analysis was done using MEME.^2^, MetaPlotR software (Olarerin-George and Jaffrey, 2017) was used to map the distribution of methylation peaks in each sample on metagene. Moreover, meRanTK software (Rieder et al., 2016) was used to analyze the conversion rate of RNA-Bis-Seq; this could count the number of effectively covered C (and mutated C), the coverage of each C, and the number of Cs converted to Ts.

For the hypermethylated m^5^C transcripts in SLE samples, transcripts with differences in mean m^5^C level ≥ 0.05 between the groups (SA and HC; SM-MA and HC) were considered to be statistically significant. To further explore the critical role of m^5^C modification in SLE, transcripts were separated into four groups based on whether the modifications resulted in hyper- or hypomethylation of m^5^C, and based on the up- or down-regulation of gene expression. Only the intersection of significant hyper- or hypomethylated transcripts, as well as significant DEGs (| FC| > 1.2, FDR < 0.05), was subjected to downstream analysis (Chen X. et al., 2019). Metascape^3^ is an effective and efficient tool for experimental biologists to comprehensively analyze and interpret OMICs-based studies in the big data era (Zhou et al., 2019). For the list of hyper-/hypomethylated genes, pathway and process enrichment analysis was carried out using the following ontology sources: Kyoto Encyclopedia of Genes and Genomes Pathway, Gene Ontology Biological Processes, Reactome Gene Sets, Canonical Pathways, and CORUM. All genes in the genome were used as the enrichment background. Terms with *p*-value < 0.01, minimum count of 3, and enrichment factor > 1.5 (ratio between the observed counts and the counts expected by chance) were collected and grouped into clusters based on their membership similarities. To further capture the relationships between the terms, a subset of enriched terms was selected and rendered as a network plot, where terms with a similarity > 0.3 were connected by edges. We selected the terms with the best *p*-values from each of the 20 clusters. Moreover, for the hypermethylated genes, protein–protein interaction enrichment analysis was also carried out using the following databases: BioGrid, InWeb_IM, OmniPath. The resultant network contained the subset of proteins that form physical interactions with at least one other member in the list. If the network contained between 3 and 500 proteins, the Molecular Complex Detection (MCODE) algorithm was applied to identify densely connected network components. Reactome Pathway significant enrichment analysis was performed to analyze the Hyper-Up related gene list or potential target genes for NSUN2 using the STRING database^4^ (Szklarczyk et al., 2017). Significance was determined based on false discovery rate < 0.05.

### RT-qPCR

*NSUN2* expression levels in the CD4^+^ T cells from 45 subjects (22 patients with SLE and 23 HCs) were detected by qRT-PCR using the QuantiNova SYBR Green PCR Kit (Qiagen). All qRT-PCR reactions were carried out using an Applied Biosystems QuantStudio^TM^ 3 Real-Time PCR Instrument (Thermo Fisher Scientific Inc.). For each reaction, 1 μL of diluted cDNA was mixed with 10 μL of 2 × SYBR Green PCR Master Mix. A final volume of 20 μL was achieved by the addition of 1.4 μL forward and reverse primers (10 μmol). The conditions for PCR amplification were as follows: 95°C for 2 min, followed by 40 cycles of 95°C for 5 s and 60°C for 10 s. The specificity of the primer amplicons was tested by melting curve analysis. All samples were tested in triplicate. The data were analyzed using the comparative threshold cycle (Ct) method. *GAPDH* was used as a control, and the relative quantification of *NSUN2* in CD4^+^ T cells was calculated using the following equation: amount of target = 2^–Δct^, where ΔCt = Ct_NSUN__2_ – Ct_GAPDH_. The following gene-specific primers were used for qRT-PCR analysis: *NSUN2*: 5′-GAACTTGCCTGGCACACAAAT-3′ and 5′-TGCTAACAGCTTCTTGACGA CTA-3′, and *GAPDH*: 5′-CAGGGCTGCTTTTAACTCTGGTAA-3′ and 5′-GGGTG GAATCA TATTGGAACATGT-3′.

### Cell Culture and Western Blot

HEK293T [American Type Culture Collection (ATCC^§^), Manassas, VA, United States] and Jurcat cells were, respectively, cultured in Dulbecco’s modified Eagle’s medium and Roswell Park Memorial Institute 1640 supplemented with 10% fetal bovine serum at 37°C in a humidified atmosphere containing 5% CO_2_ (SANYO, MCO-175, Osaka prefecture, Japan). HEK293T and Jurcat cells and CD4^+^ T cells from five HCs and five patients with SLE were lysed using protein lysis buffer (Beyotime Institute of Biotechnology, Beijing, China) supplemented with protease inhibitor cocktail (Pierce, Rockford, IL, United States) at 4°C for 20 min. Protein samples were separated using 10% sodium dodecyl sulfate-polyacrylamide gel electrophoresis and then electrophoretically transferred to polyvinylidene difluoride membranes (Millipore, Billerica, MA, United States). Anti-NSUN2 antibody (20854-1-AP; Proteintech, Rosemont, IL, United States) was diluted with primary antibody dilution buffer (Beyotime Institute of Biotechnology) to 1:5,000, and anti-GAPDH antibody (GOOD HERE, Hangzhou, China) was also diluted to 1:1,000. The membranes were then washed with TBST buffer five times for 5 min each and incubated with horseradish peroxidase-conjugated goat anti-rabbit IgG secondary antibody (1:5,000 dilution) (Cell Signaling Technology, Danvers, MA, United States) for 1.5 h at 37°C. Bands were detected using enhanced chemiluminescence and visualized with a Gel Doc 2,000 (BioRad, Hercules, CA, United States).

### Statistical Analysis

Statistical analysis was performed using SPSS 22.0 software (IBM, Armonk, NY, United States) and GraphPad Prism version 8.0.1 software (GraphPad Software, La Jolla, CA, United States). An independent sample *t*-test was performed to analyze the difference of the mRNA expression levels in CD4^+^ T cells of patients with SLE and HCs; the data are presented as the means ± standard deviation. A non-parametric Mann–Whitney *U*-test was performed to analyze the difference of the mRNA m^5^C methylated levels or protein expression levels in CD4^+^ T cells of patients with SLE and HCs; the data are presented as 50% quantile (25% quantile, 75% quantile). A two-sided *p* < 0.05 was considered to represent a statistically significant difference.

## Results

### mRNA Methylation Profiling of HCs and Patients With SLE Presenting Diverse Disease Activity

**FIGURE 1:**
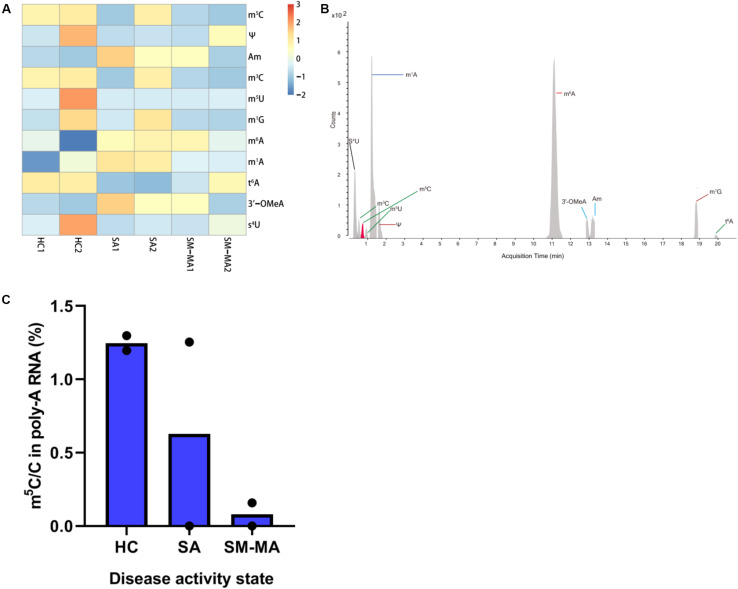
Detection of mRNA modifications by LC-MS/MS among healthy controls (HCs) and systemic lupus erythematosus (SLE) patients with different disease activity. **(A)** Heatmap of normalized abundance (modification/canonical nucleotide) of 11 mRNA modifications detected by LC-MS/MS between HCs and SLE patients. Red indicates a high *z*-score, whereas blue indicates a low *z*-score. **(B)** LC-MS/MS extracted ion chromatograms of modified nucleotides analyzed in CD4^+^ T cells mRNA of HCs and SLE patients. S_4_U, 4-thiouridine; m^3^C, 3-methylcytosine; m^5^C, 5-methylcytosine; m^5^U, 5-methyluridine; m^1^A, N^1^-methyladenosine; Ψ, pseudouridine; m^6^A, N6-methyladenosine; 3′-OMeA, 3’-O-methyladenosine; Am, 2’-O-methyladenosine; m^1^G, 1-methylguanosine; t_6_A, N6-threonylcarbamoyladenosine. **(C)** Global dynamics of calibrated m^5^C/C levels in HCs and SLE patients with different disease activity (SA, SM-MA). Bars show the mean of individual biological replicates (*n* = 2).

We isolated mRNA from the CD4^+^ T cells of 10 patients with SLE exhibiting stable activity (SA group), 10 patients with moderate/major activity (SM-MA group), and 18 HCs (HC group), then combined equal amounts of mRNA from 5 or 9 individuals, respectively, into one pool for each group. Finally, each group consisted of two separate pools for analysis. We generated mRNA methylomes for six separate pools using LC-MS/MS and identified that the mRNA levels were differently modified between HCs and patients with SLE exhibiting diverse disease activity (Figures 1A,B and Supplementary Table S2). A total of 11 modifications (including m^5^C, Ψ, m^6^A, and m^1^A) previously identified in mRNA were detected in our study among these groups. Compared with those of HCs, the Am, 3′OMeA, m^1^A, and m^6^A levels in CD4^+^ T cells of SLE were elevated, whereas those of m^5^C, Ψ, m^3^C, m^1^G, m^5^U, and t^6^A were decreased (Figure 1A and Supplementary Figure S1). As it has been reported that m^5^C is a newly discovered internal mRNA modification in eukaryotes (Amort et al., 2017) that regulates immune response including oncogene activation (Chen X. et al., 2019), in this study, we further focused on the m^5^C level in overall mRNA. Compared to those in HCs, the m^5^C/C levels in CD4^+^ T cells were markedly lower in both SA and SM-MA groups (Figure 1C). Moreover, the m^5^C/C levels in CD4^+^ T cells were decreased in patients with SLE exhibiting increasing disease activity.

### Distribution Profiling of m^5^C in mRNA of Patients With SLE Exhibiting Different Disease Activity and HCs

**FIGURE 2:**
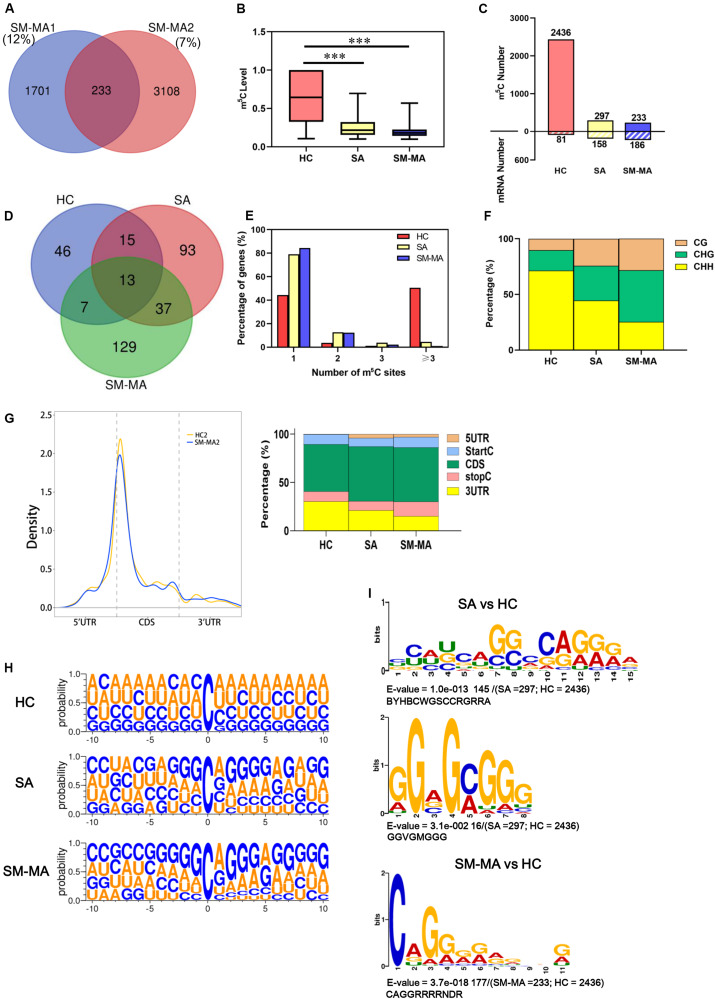
Distribution profiles of m^5^C in mRNAs from systemic lupus erythematosus CD4^+^ T cells. **(A)** Venn diagram showing overlap of m^5^C sites within mRNAs between two SLE moderate/major active (SM-MA) pool replicates. **(B)** Bar chart showing level of m^5^C sites among healthy controls (HCs) and SLE stable (SA) and SLE moderate/major active (SM-MA) patients (***p < 0.001). **(C)** Bar chart showing number of m^5^C sites and m^5^C-containing mRNA among HCs and SA and SM-MA patients. **(D)** Venn diagram showing overlap of genes within m^5^C-containing mRNAs in HCs and SA and SM-MA patients. **(E)** Proportion of genes harboring different numbers of m^5^C sites in three groups. The majority of genes harboring only one m^5^C site except that in the HC group. **(F)** Bar chart showing percentage of mRNA m^5^C sites identified in each sequence context (CG, CHG, and CHH, where H = A, C, or U) in CD4^+^ T cells of HCs and SA and SM-MA patients. **(G)** Distribution of m^5^C sites along mRNA transcripts. The moving averages of percentages of mRNA m^5^C sites are shown (raw data; only HC2 and SM-MA2 group are shown here) (**left**). The averages of percentages of mRNA m^5^C sites within 5′UTR, StartC, CDS, stopC, and 3′UTR regions in transcriptomes are shown. Distribution of m^5^C sites peaks along mRNA transcripts are shown (**right**). **(H)** Sequence frequency logo for the sequences proximal to mRNA m^5^C sites among HCs and SLE patients. **(I)** Top differential enrichment mode motifs (SA vs. HC; SM-MA vs. HC) enriched across the sequences proximal to mRNA m^5^C sites identified from HCs and SA and SM-MA patients (only *E*-value < 0.05 was shown here).

To obtain a transcriptome-wide landscape of m^5^C profiling, we further performed mRNA Bis-Seq analysis on mRNA samples purified from CD4^+^ T cells of patients contributing to the SA, SM-MA, and HCs pools according to a recently described study (Yang et al., 2017). The overlapping m^5^C sites in two independent pools from each group were selected for follow-up analysis. For example, a total of 233 m^5^C sites identified in both SM-MA patient replicates (“high-confidence” set) were used in subsequent bioinformatics analyses (Figure 2A and Supplementary Table S3). Overall, the m^5^C levels (approximately 62.8%) in mRNA of CD4^+^ T cells of HCs were considerably higher compared with those from both SA and SM-MA groups (Figure 2B), as determined by LC-MS/MS analyses. Furthermore, the overall m^5^C level in mRNA of CD4^+^ T cells from the SM-MA group (19.6%) was relatively lower than that in the SA group (25.3%). Notably, the number of m^5^C-modified mRNA molecules exhibited opposite changes to the number of m^5^C-modified sites with increasing disease activity (Figure 2C). Among the m^5^C sites/mRNAs identified in CD4^+^ T cells of SA and SM-MA groups, more m^5^C-containing gene transcripts were observed with fewer m^5^C methylation sites (297/158 and 233/186, respectively) within mRNAs (Figure 2C), which is contrary to the results in CD4^+^ T cells of HCs (2436/81).

A Venn diagram was used to analyze the overlap of genes within m^5^C-containing mRNAs in HC, SA, and SM-MA groups. An overlap of 13 m^5^C mRNAs between the groups was found as shown in Figure 2D. Additionally, 46 m^5^C-containing mRNAs were detected in only HCs, 93 only in the SA group, and 129 only in the SM-MA group. Next, the proportion of genes harboring different numbers of m^5^C sites in the three groups was further analyzed. Compared with that of HCs, the percentage of m^5^C-containing gene transcripts harboring one m^5^C site was obviously higher in CD4^+^ T cells of SA and SM-MA groups (44.4% vs. 79.1% and 84.4%, respectively), whereas the percentage of transcripts harboring three m^5^C sites was significantly lower (50.6% vs. 4.4% and 1.1%) (Figure 2E). In turn, the m^5^C-containing gene transcript percentage containing two or three m^5^C sites in SLE was slightly higher than that in HCs. Moreover, the percentage of mRNA m^5^C sites identified in each sequence context (CG, CHG, and CHH, where H = A, C, or U) in HCs and patients with SLE exhibiting different disease activity markedly differed, with an increase in CG and CHG regions, respectively, from 10.2% and 18.3% in HCs to 24.2% and 31.3% in the SA group, further increasing to 28.3% and 46.4% in the SM-MA group; conversely, a sharp decrease was observed in CHH regions from 71.4% in HCs to 44.4% in SA, then to 25.3% in SM-MA groups (Figure 2F).

We next determined the enrichment feature of m^5^C sites in mRNA transcripts. m^5^C peaks were divided into 5′-untranslated region, StartC, coding sequence (CDS), StopC, and 3′untranslated regions according to their locations in RNA transcripts (Figure 2G). Notably, m^5^C sites were also enriched in startC and CDS regions immediately downstream of translation initiation sites (Figure 2G), which is similar to previous observation in HeLa cells (Yang et al., 2017). Similarly, a sequence frequency logo identified that m^5^C sites were embedded in CG-rich environments (Figure 2H). The most significantly enriched motifs in patients with SLE and HCs were further analyzed and presented in Supplementary Figure S2. Moreover, the top differential enrichment mode motifs (SA vs. HC; SM-MA vs. HC) enriched across the sequences proximal to mRNA m^5^C sites were further identified (Figure 2I). These were BYHBCWGSCCRGRRA (*p* = 1.0e−013) and GGVGMGGG (*p* = 3.1e−002) between SA and HC groups, which were present in approximately 49 and 5% of the methylated sequences proximal to mRNA m^5^C sites, respectively (Figure 2I). Between SM-MA and HC groups, CAGGRRRRNDR (> 75% of the methylated sequences proximal to mRNA m^5^C sites) was most significantly enriched (*p* = 3.7e−018) (Figure 2I), which indicated that CCRGRRA and CAGGRR might constitute the specific motif of m^5^C-modified genes in SA and SM-MA groups, respectively.

### Pathway Analysis of the Genes Containing Significantly Altered m^5^C Sites (Differentially Methylated Genes) Between Patients With SLE and HCs

**FIGURE 3:**
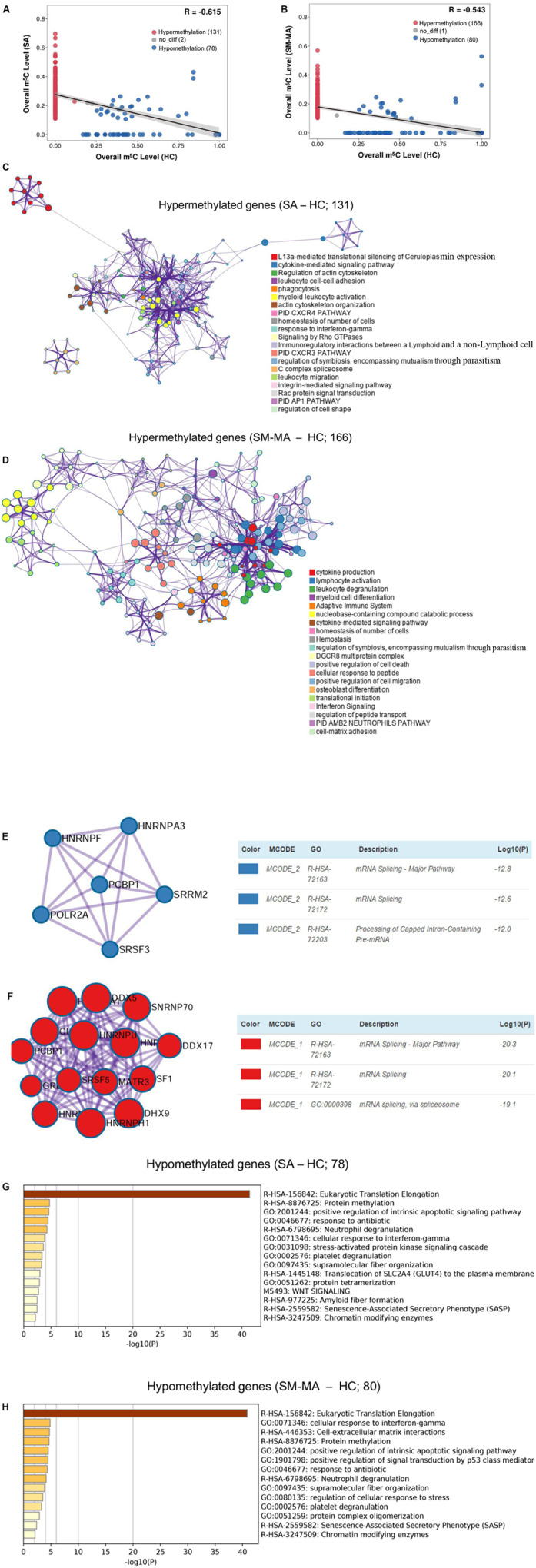
Pathway analysis of genes containing significantly altered m^5^C sites (differentially methylated genes, DMGs) between healthy controls (HCs) and systemic lupus erythematosus (SLE) patients. Correlation of m^5^C methylation between SLE stable (SA) **(A)** or SLE moderate/major active (SM-MA) **(B)** group with HC group. Regression lines and 95% confidence intervals (shaded regions) are shown. Spearman’s correlation coefficients (*R*) were calculated using the “cor.test” function in the statistical language R. Hypermethylation (red points) indicates that the m^5^C transcripts with altered methylation level in SLE patients is higher than that in HCs. Hypomethylation (blue points) indicates that the m^5^C transcripts with altered methylation level in SLE patients are lower than that in HCs. **(C,D)** Network of enriched terms colored by cluster ID for differentially hypermethylated genes (**C**, SA vs. HC; **D**, SM-MA vs. HC). Each term is represented by a circle node, where its size is proportional to the number of input genes that are related to that term, and its color represents its cluster identity. Terms with a similarity score > 0.3 are linked by an edge (thickness of the edge represents the similarity score). **(E,F)** The molecular complex detection (MCODE) components identified for the differentially hypermethylated genes (**E**, SA vs. HC; **F**, SM-MA vs. HC). Pathway and process enrichment analysis has been applied to each MCODE component independently, and the three best-scoring terms according to *p*-value have been retained as the functional description of the corresponding components. **(G,H)** Bar graph of enriched terms across the differentially hypomethylated genes (**E**, SA vs. HC; **F**, SM-MA vs. HC), colored according to *p*-values.

Accumulating evidence has shown that numerous mRNAs with m^5^C methylation promote the pathogenesis of human diseases such as urothelial carcinoma (Chen X. et al., 2019). To provide functional insights regarding whether mRNAs carrying m^5^C methylation in CD4^+^ T cells are linked to SLE, we explored the overall changes of transcript m^5^C methylation by mapping the m^5^C distribution in CD4^+^ T cells through mRNA-Bis-Seq data between SA/SM-MA and HC groups. We found 78 m^5^C hypomethylated transcripts and 131 m^5^C hypermethylated transcripts in CD4^+^ T cells of the SA group compared to those in HCs (Figure 3A and Supplementary Table S4). Moreover, compared to HCs, 80 transcripts with decreased m^5^C levels and 166 transcripts with increased m^5^C levels were found in CD4^+^ T cells of the SM-MA group (Figure 3B and Supplementary Table S4).

Additionally, we further investigated the functions of differentially methylated genes in patients with SLE using Metascape. m^5^C hypermethylated genes in both SA and SM-MA groups were found to be significantly enriched in immune-related pathways, including cytokine-mediated signaling pathway and homeostasis of number of cells (Figures 3C,D and Supplementary Table S5), which consistent with the function of m^5^C-modified transcripts in HEK293 cells (Sun et al., 2019). Among these, MCODEs analyses of hypermethylated genes in SA/SM-MA groups for the top pathways and process enrichment result identified mRNA splicing in the protein–protein interaction networks of both SA versus HC and SM-MA versus HC (Figures 3E,F), which is similar to the results of a previous study (Yang L. et al., 2019). Gene ontology enrichment analysis was also performed for m^5^C hypomethylated transcripts between SA/SM-MA and HC groups. Overall, m^5^C hypomethylated transcripts in SA or SM-MA groups were found to be significantly enriched in eukaryotic translation elongation and protein methylation (Figures 3G,H and Supplementary Table S6).

### Functional Enrichment of Differentially Expressed Genes (DEGs) Between Patients With SLE and HCs

As shown in Supplementary Figure S3A, principal component analysis results of mRNA profiles indicated that the gene expression patterns were similar within the same group, however, significantly different between HC, SA and SM-MA groups. In SA versus HCs and SM-MA versus HCs, gene ontology analysis showed that the DEGs in both groups were significantly enriched in some essential molecular functions (e.g., protein binding and chemokine activity), cellular components (e.g., plasma membrane, cytosol and extracellular region and space), and biological processes (e.g., immune response, innate immune response, cytokine-mediated signaling pathway, and type I interferon signaling pathway) (Supplementary Figure S3B), which was similar to our previous study on peripheral blood mononuclear cells of SLE (Guo et al., 2018). Moreover, pathway analysis of DEGs in the SA versus HC and SM-MA versus HC groups showed that DEGs in both comparisons were linked to inflammatory pathways and immune-related pathways in organismal systems (e.g., chemokine signaling, Toll-like receptor signaling, and IL-17 signaling pathway), human disease (e.g., rheumatoid arthritis and SLE), and environmental information processing (e.g., cytokine–cytokine receptor interaction and TNF-signaling pathway) (Supplementary Figure S3C).

### Integration Analyses of m^5^C-Containing mRNA Methylation and mRNA Transcript Expression

**FIGURE 4:**
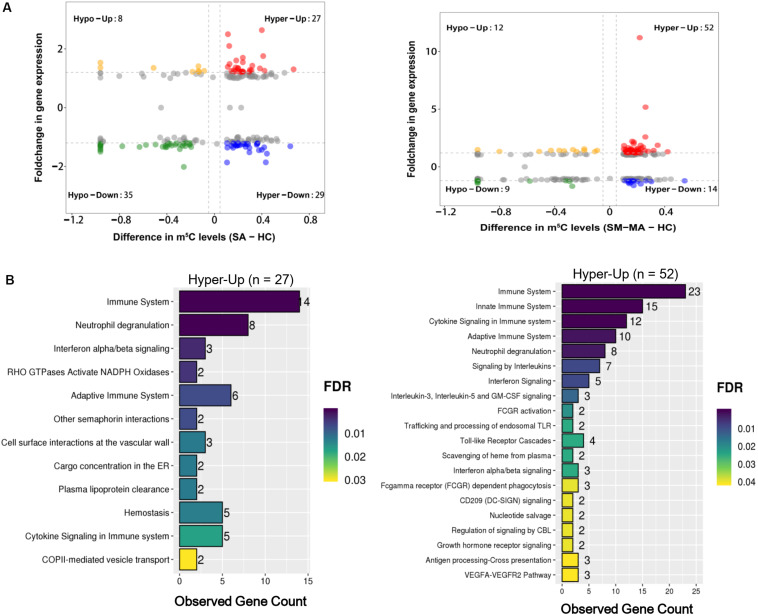
Conjoint analysis of differentially methylated genes and differentially expressed genes. **(A)** Distribution of mRNAs with a significant change in both m^5^C modification and gene expression levels in healthy controls (HCs) and SLE stable (SA)/SLE moderate/major active (SM-MA) patients, respectively. **(B)** Reactome pathways analysis of hypermethylated mRNAs with up-regulated genes in SA versus HC (**left**) or SM-MA versus HC (**right**) groups, respectively.

As previously reported, the up-regulation of numerous oncogene RNAs with hypermethylated m^5^C sites has been causally related to human bladder cancer (Chen X. et al., 2019). Here, we further explored the association between RNAs with m^5^C modification and mRNA transcript expression by integrated RNA-Seq and RNA-Bis-Seq data. We found no strong correlation between m^5^C-containing mRNA methylation and mRNA transcript expression levels in patients with SLE (Figure 4A), which was consistent with previous report (Wang et al., 2019). Moreover, mRNA m^5^C modification has been reported to up-regulate mRNA expression by stabilizing mRNA (Chen X. et al., 2019). Toward this end, we also identified 27 and 52 up-regulated expressed mRNAs exhibiting hypermethylated m^5^C in the CD4^+^ T cells of SA versus HCs and SM-MA versus HC groups, respectively (Figure 4A and Supplementary Table S7). Notably, ADAR (Roth et al., 2018), an SLE-related gene, was identified in these data as exhibiting both m^5^C hypermethylation and mRNA up-regulation in patients with SLE (Supplementary Table S7). Moreover, we further investigated the enrichment of signaling pathways of up-regulated expressed RNAs exhibiting hypermethylated m^5^C levels using Reactome. In particular, these transcripts were enriched prominently in categories of immune system, neutrophil degranulation, cytokine signaling in immune system, and interferon signaling (Figure 4B and Supplementary Table S8), which indicated that up-regulated genes with high m^5^C-levels modification participate in the flares and remission of patients with SLE through disruption of the patient’s immune system.

### Expression Level and Regulation of NSUN2 in CD4^+^ T Cells From Patients With SLE

**FIGURE 5:**
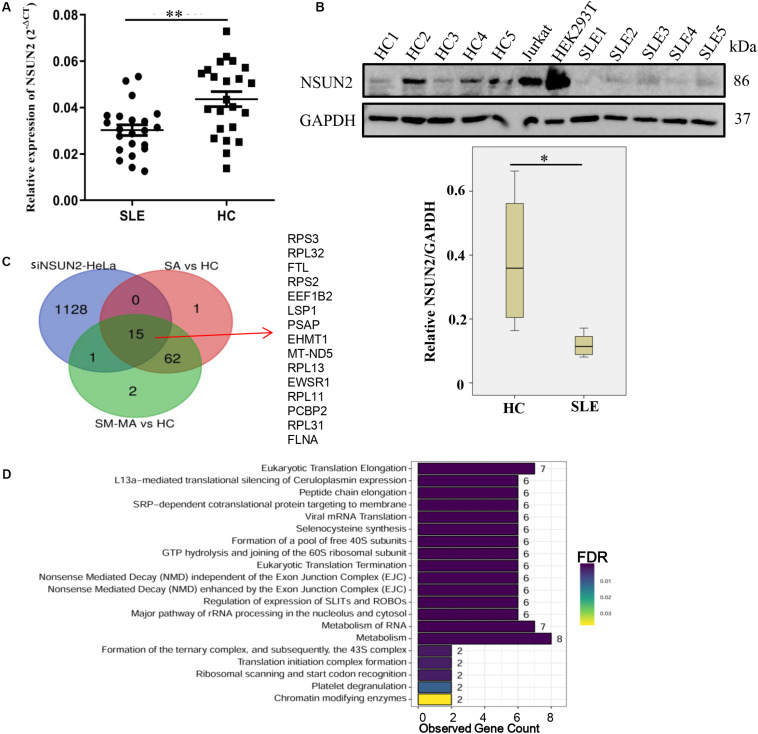
Down-regulation of NSUN2 is involved in the pathogenesis of systemic lupus erythematosus. **(A)** NSUN2 mRNA expression level in CD4^+^ T cells from SLE patients (*n* = 22) and healthy controls (HCs) (*n* = 23). Data are presented as 2^–Δct^ relative to GAPDH expression (mean ± standard deviation; **p < 0.01). **(B)** NSUN2 protein expression levels in CD4^+^ T cells from five SLE patients, five HCs, and two positive control cell lines (Jurkat and HEK293T). The gray scale of WB was measured by ImageQuant software and compared between SLE patients and HCs (*p < 0.05). NSUN2 protein expression was normalized to that of GAPDH. **(C)** Overlap of differentially hypomethylated genes in siNSUN2-HeLa cells with SLE stable (SA) versus HC and SLE moderate/major active (SM-MA) versus HC hypomethylated mRNAs. **(D)** Reactome pathways analysis of potential methylated target genes of methylase NSUN2.

As NSUN2 is the only known mRNA m^5^C methyltransferase (Li et al., 2017), we further explored the expression level of NSUN2 in CD4^+^ T cells. Compared with that in HCs, its mRNA and protein expression in CD4^+^ T cells from patients with SLE was significantly decreased (*p* < 0.01 and *p* < 0.05, respectively) (Figures 5A,B). Similar to the down-regulated m^5^C methylation levels in transcripts observed in siNSUN2-HeLa cells (Yang et al., 2017), we also identified 15 m^5^C hypomethylated transcripts in both SA versus HC and SM-MA versus HC groups (Figure 5C). Reactome pathways analysis revealed that these 15 transcripts were mainly enriched in representative transcription-related pathways including eukaryotic translation elongation and termination, peptide chain elongation, and mRNA translation and metabolism (Figure 5D), which indicted that these abnormally hypomethylated transcripts likely contribute to the risk of developing SLE.

**FIGURE 6:**
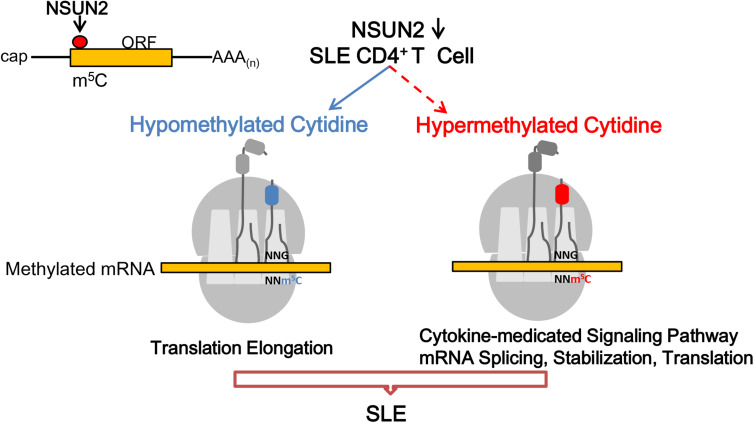
Proposed model for the landscape of NSUN2-mediated regulation of m^5^C methylation in the pathogenesis of systemic lupus erythematosus. m^5^C formation is promoted by NSUN2. Genes with dysregulated m^5^C methylation participate in the flares and remission course of SLE.

As suggested in the model shown in Figure 6, NSUN2-down-regulated expression in SLE contributes to the down-regulation of m^5^C levels. Among the affected transcripts, hypermethylated m^5^C transcripts in CD4^+^ T cells of patients with SLE mainly participated in the cytokine-related signaling pathway, and mRNA splicing, stabilization, and translation, whereas hypomethylated m^5^C transcripts were mainly involved in translation elongation in the pathogenesis of SLE.

## Discussion

Recent studies have identified that DNA hypomethylation might contribute to the immune response and over-reactivity in SLE CD4^+^ T cells (Zhang et al., 2013; Zhao et al., 2014; Yeung et al., 2019). In addition, biochemical modifications to mRNA, especially m^6^A and m^5^C, have also been shown to be of considerable importance with regard to the biological functions of developing disease (Li et al., 2018; Chen X. et al., 2019). As a newly discovered methylation modification in eukaryotic mRNAs, m^5^C methylation was reported as an unprecedented mechanism of regulating oncogene activation in bladder carcinoma (Chen X. et al., 2019). Nevertheless, the regulation and biological role of m^5^C modification in SLE pathogenesis remains unclear. Here, we displayed transcriptome-wide m^5^C modification profiles of SLE CD4^+^ T cells with different disease activity, investigating gene expression and disease-related pathways. As the number of CD4^+^ T cells able to be isolated from the peripheral blood is limited and patients with SLE exhibit heterogeneity, we pooled RNA of CD4^+^ T cells from 5/9 subjects equally into a single pool. As reported previously, for epigenetic signs, RNA pooling allowed the effective focus on actual epigenetic patterns under the background of the numerous random inter-individual differences (Docherty et al., 2009; Toperoff et al., 2012; Zhao et al., 2014). Additionally, sample pooling ensures the ability to obtain multi-omics information from the same samples and precise detection of average RNA methylation and expression levels in large groups of mRNAs from individual patients. And PCA results of mRNA profiles indicated that pooled samples are valid for the analysis of methylation profiling.

In this study, we revealed the modifications in global methylated mRNA in SLE CD4^+^ T cells for the first time, illuminating multiple patterns of mRNA modification in SLE. We also uncovered the primary differences of m^5^C modifications including prevalence, number, and unique distribution along transcripts in HCs and patients with SLE. Notably, we identified that m^5^C levels were decreased in CD4^+^ T cells of patients with SLE compared with those of HCs along with lower m^5^C levels associated with severe disease activity in patients with SLE. Consistent with DNA hypomethylation in SLE, the results also indicated that m^5^C hypomethylation in SLE CD4^+^ T cells might increase T cell self-reactivity in patients with SLE. In comparison, a recent study reported that the levels of DNA hydroxymethylation, transited from DNA m^5^C, were increased and some genes exhibited promoter region hypomethylation in CD4^+^ T cells from patients with SLE (Zhao et al., 2016), which may explain the potential mechanism of m^5^C in SLE. Notably, in SLE patients, we observed increased m^5^C-containing transcripts with low modification levels in CD4^+^ T cells, primarily containing one or two m^5^C sites, among which certain mRNAs were determined to likely be involved in SLE pathogenesis. It should be noted that the overall modification level of one sample is primarily determined by the number of m^5^C modified transcripts in that sample, as well as the level of m^5^C modification of each transcript. Therefore, an increase in the number of m^5^C modified transcripts in one sample does not necessarily indicate an overall higher level of modified m^5^C.

Notably, our analysis of the sequences proximal to mRNA m^5^C sites revealed several hitherto unknown features of m^5^C distribution in SLE mRNA. First, m^5^C sites are located in the vicinity of the translation start sites of mRNAs, and these patterns of distribution are highly conserved in patients with SLE. A similar pattern of distribution of m^5^C sites has been reported in HeLa cells (Yang et al., 2017), cancers (Chen X. et al., 2019), and mouse embryonic stem cells and brain tissues (Amort et al., 2017), whereas the patterns differ in *Arabidopsis* (Cui et al., 2017). Second, m^5^C sites were observed to be enriched in the GC-rich region with the highest enrichment motifs of m^5^C sites being CCRGRRA and CAGGRR. This may be relevant as the regulatory effects of RNA modification are usually interpreted by their reader proteins (Roundtree et al., 2017). However, to date, only three known m^5^C binding proteins are known to recognize the m^5^C modified mRNAs: the nuclear ALYREF promotes mRNA export (Yang et al., 2017), and the cytoplasmic YBX1 and TRM4B maintain mRNA stability in mammals and plants (Cui et al., 2017; Chen X. et al., 2019). Moreover, the most enriched motifs for m^5^C methylated peak summits in *Arabidopsis thaliana* were HACCR and CTYCTYC (Cui et al., 2017), which different from those in patients with SLE. The specific motifs in these patients implied the presence of other as-yet unknown potential reader(s), which needs to be validated though additional study. Nevertheless, these distribution features, together with the observation of low m^5^C levels in mRNA with high disease activity, imply that m^5^C modification in CDSs may play a crucial role in mediating the flares and remission of SLE.

In additional, compared with the HC group, we identified 209 (including 131 hypermethylated and 78 hypomethylated transcripts) and 246 (including 131 hypermethylated and 78 hypomethylated transcripts) dysregulated m^5^C-methylated transcripts in the SA and SM-MA group, respectively. These abnormally methylated transcripts in SLE CD4^+^ T cells might contribute to SLE pathogenesis. CD4^+^ T cells constitute the principle components of the adaptive immune system that can secrete cytokines; moreover, naive CD4^+^ T cells can differentiate into a variety of different cell subsets. However, dysregulation of CD4^+^ T cell differentiation is crucial to the pathogenesis of SLE. Increased TGF-β and IL-6 serve as major contributors to the imbalance of Th17 and regulatory T cell differentiation (Raphael et al., 2015), which promotes SLE development. Consistent with this, our transcription-wide analysis identified transcripts with increased m^5^C in patients with both stable and active SLE as being involved in several important inflammatory biological process and immune-related pathways including the cytokine-mediated signaling pathway, homeostasis of number of cells, and interferon response signaling, which suggested that transcripts exhibiting m^5^C hypermethylation may play a very vital role in the pathogenesis of SLE. For example, cytokines (such as interleukin-6, interleukin-10, and tumor necrosis factor) in patients with SLE can contribute to SLE susceptibility through the promotion of autoantibody production and inflammation (Tsokos et al., 2016). Moreover, in this study, although the methylated cytosine depth of both transforming growth factor B1 and interleukin-6 receptor transcripts were < 5, both were m^5^C hypermethylated in CD4^+^ T cells of SA/SM-MA groups, which might stabilize their mRNA expression and lead to the imbalance of Th17/regulatory T cell differentiation and, thus, likely contribute to SLE pathology.

Interferon signaling has also been reported to constitute a crucial driver of the development of autoimmunity in lupus mice (Ikeda et al., 2017; Zeng et al., 2019) and demonstrates close association with SLE disease activity in human (Ronnblom and Elkon, 2010). Furthermore, homeostasis of the number of cells and the immune system is also responsible for the development of autoimmune diseases (Saferding and Bluml, 2019). In addition, a common major pathway of hypermethylated m^5^C transcripts is mRNA splicing. Recently, growing evidence has supported differential splicing of mRNAs as a mechanism in T cells (Schaub and Glasmacher, 2017) that is linked to presentation of immune-related diseases, considering that some transcripts with decreased m^5^C methylation level were significantly enriched in eukaryotic translation elongation. As inflammation is controlled by transcriptional regulation including the pathogen-associated molecular process of mRNA translation elongation (Bianco et al., 2019), these findings suggest that m^5^C modification may serve to remodel the transcriptional regulation in patients with SLE.

As reported previously, m^5^C-containing transcript modification and mRNA expression levels show no strong correlation in bladder cancers (Chen X. et al., 2019), as was also observed in patients with SLE in this study. Through integration with our previous data (Guo et al., 2018), we found that some dysregulated mRNAs likely participate in the immune system in either peripheral blood mononuclear cells or CD4^+^ T cells, whereas some transcripts significantly enriched in chemokine activity processes were detected only in CD4^+^ T cells. This may be due to the distinctions in disease phenotype in the sample pool, sample size, and sample type. As mRNA m^5^C modifications may lead to up-regulated mRNA expression by stabilizing mRNA in the immune system (Chen X. et al., 2019), we focused on up-regulated transcripts with increased m^5^C levels. We identified that significantly up-regulated transcripts exhibiting hypermethylated m^5^C levels in patients with both stable and active SLE were associated with SLE-related biological process and immune-related pathways, such as the immune system, cytokine signaling pathway, and interferon signaling. These pathways are the same as the major pathways in transcripts with increased m^5^C modification are involved, indicating that up-regulated transcripts with increased m^5^C levels may play a central part in the flares of patients with SLE. However, further functional research will be needed to clarify the functional connection between RNA m^5^C modification and gene expression in the development of SLE.

Recently, m^5^C methylation in eukaryotic cells has been identified to largely depend on the function of the known “writer” NSUN2, which functions as an mRNA methyltransferase (Bohnsack et al., 2019; Yang Y. et al., 2019). Moreover, it was reported that NSUN2 affects cell biological processes including cell proliferation, migration, and mRNA metabolism by regulating mRNA m^5^C modification (Wang et al., 2011; Courtney et al., 2019). Compared to that in HCs, the NSUN2 expression (including mRNA and protein) in CD4^+^ T cells of patients with SLE was significantly lower, which further confirmed the Bis-Seq data of overall m^5^C levels in SLE. Consistent with our results, Han et al. (2020) found NSUN2 up-regulation, as well as aberrant mRNA m^5^C gain and loss in PM2.5-induced pulmonary fibrosis mouse models. This phenomenon has also been described in bladder cancer and Zebrafish (Chen X. et al., 2019; Yang Y. et al., 2019). In summary, the results for aberrant methylated modifications, as determined by RNA-Bis-Seq, are in part due to the down-regulation of NSUN2; however, we cannot rule out the presence or role of other m^5^C methyltransferases in SLE patients resulting in the generation of more m^5^C-containing transcripts. Additionally, this provided indirect evidence that the abnormal NSUN2 may constitute a good target associated with m^5^C modification in SLE CD4^+^ T cells. Through comparison with the RNA-Bis-Seq data of NSUN2-knockdown HeLa cells (Yang et al., 2017), we found that 15 transcripts were similarly m^5^C hypomethylated in CD4^+^ T cells of SLE, the majority of which primarily participated in eukaryotic translation elongation and termination and RNA metabolism. This observation was consistent with previous studies (Dominissini and Rechavi, 2017; Yang et al., 2017; Chen X. et al., 2019), indicating that the majority of core hypomethylated m^5^C genes in SLE are influenced by NSUN2 expression. However, to some extent, CD4^+^ T cells and HeLa cells differ in the aspects of molecular function, biological process, and diseases models. The relationship between NSUN2-mediated RNA methylation and gene expression in patients with SLE is still unclear. Further studies on immune cells and animal models will be required to obtain a common understanding of the role of NSUN2 in patients with SLE.

Moreover, based on the integrated analysis of m^5^C-containing mRNA methylation and mRNA transcript expression, 5-methylcytosine is expected to be confirmed as a potential and pivotal modification in the pathogenesis of SLE. Consequent to NSUN2 dysregulated expression, m^5^C methylation levels were down-regulated in SLE. In these patients, RNAs exhibiting m^5^C hypomethylation, enriched in translation initiation regions, participated in translation elongation, whereas hypermethylated mRNAs were involved in SLE-related and cytokine-mediated signaling pathways, and mRNA splicing, stabilization, and translation. It has been found that several mRNA m^6^A methyltransferases (including METTL3/14, WTAP, RBM15/15B, etc.) are relevant to installation of m^6^A modification in mRNA (Chen X.Y. et al., 2019). In addition to NSUN2, we speculated that other writer(s) might exist and regulate m^5^C modification in SLE, resulting in the generation of more m^5^C-containing transcripts. However, the small sample size and heterogeneous sample pools constituted limitations in this study. For example, although a large proportion of the modifications exhibited no distinct difference in the global methylation profile of the HC group, several modifications (Ψ, m^5^U, m^1^G and s^4^U) were found to differ from each other. Subsequent studies using larger sample sizes will be needed to validate these overall modification levels in SLE. In addition, considering that the most abundant modification was that of m^6^A, its mRNA should be jointly analyzed with m^5^C in the context of SLE pathogenesis.

## Conclusion

In summary, this study presented the first transcriptome-wide m^5^C methylated map of CD4^+^ T cells from patients with SLE and HCs. Our results offered a potential link between abnormal m^5^C RNA modifications and immune-related pathways. This novel epigenetic modification may provide a different perspective for better understanding the pathogenesis of SLE. Moreover, targeting m^5^C modifications is likely to become a therapeutic strategy in SLE in the future.

## Data Availability Statement

The raw data supporting the conclusions of this article will be made available by the authors, without undue reservation, to any qualified researcher.

## Ethics Statement

The studies involving human participants were reviewed and approved by the Medical Ethical Committees of The First Affiliated Hospital of Wenzhou Medical University. The patients/participants provided their written informed consent to participate in this study.

## Author Contributions

GG and HW performed the experiments, analyzed and interpreted the data, and drafted the manuscript. XS performed the experiments and statistical analysis. LY, KY, and ZC acquired the data and provided material support. HZ, ZJ, and XX contributed to the conception and design of the study, and analyzed and interpreted the data, supervised the study, provided the project funding, revised the manuscript, and finally approved the version of the manuscript for publication. All authors read and approved the final manuscript.

## Conflict of Interest

The authors declare that the research was conducted in the absence of any commercial or financial relationships that could be construed as a potential conflict of interest.

## Footnotes

1 http://weblogo.threeplusone.com/

2 http://meme-suite.org/

3 http://metascape.org/gp/index.html

4 https://string-db.org/

## Supplementary Material

FIGURE S1 Global dynamics of mRNA modifications (calculated as ratio of modified/unmodified nucleotide). The maximal ratio of each modification was set to 1. Points represent individual biological replicates (*n* = 2) with bars showing the mean.

FIGURE S2 Top classic mode motifs enriched across the sequences proximal to mRNA m^5^C sites identified from healthy controls and SLE stable and SLE moderate/major active patients, respectively (only *E*-value < 0.05 is shown here).

FIGURE S3 Pathway analysis of differentially expressed mRNAs in the CD4^+^ T cells from healthy controls (HCs) and systemic erythematosus lupus patients (SLE). **(A)** Principal component analysis (PCA) of the mRNA expression profiles in CD4^+^ T cells from HCs and SLE patients. This is another way to visualize sample-to-sample distances. In this ordination method, the data points are projected onto the 2D plane such that they spread out in the two directions that explain most of the differences. The *x*-axis separates the data points the most. The values of the samples in this direction are referred to as PC1. The *y*-axis (it must be orthogonal to the first direction) separates the data points the second most. The values of the samples in this direction are referred to as PC2. The percent of the total variance that is contained in the direction is shown on the axis label. Note that these percentages do not add to 100%, because there are more dimensions that contain the remaining variance (although each of these remaining dimensions will explain less than the two that we see). This analysis was performed using R language. **(B)** Gene ontology analysis of differentially expressed mRNAs. Red indicates molecular function (MF), green indicates cellular component (CC), and blue indicates biological process (BP). **(C)** Pathway analysis of differentially expressed mRNAs. Red indicates organismal systems, green indicates human diseases, and blue indicates environmental information processing.

TABLE S1 Clinical features for systemic lupus erythematosus (SLE) patients included in our study.

TABLE S2 Multiple-reaction monitoring table of mRNA modifications.

TABLE S3 Dataset of all identified m5C sites in CD4+ T cells of HCs and SLE patients.

TABLE S4 Genes with the differential m5 C levels in SLE CD4+ T cells compared to healthy controls.

TABLE S5 The enriched go terms in genes with differential RNA hypermethylation in SLE CD4+ T cells compared with healthy controls according to metascape analysis.

TABLE S6 The enriched go terms in genes with differential RNA hypomethylation in SLE CD4+ T cells compared with healthy controls according to metascape analysis.

TABLE S7 Increased m5C levels and up-regulated expression in SLE CD4+ T cells compared with healthy controls.

TABLE S8 Reactome pathways enriched in genes with increased m5C levels and up-regulated expression in SLE CD4+ T cells compared with healthy controls.
