# Supplementary figures and images for: Disease Activity-Associated Alteration of mRNA m5 C Methylation in CD4+ T Cells of Systemic Lupus Erythematosus
Source: Front Cell Dev Biol. 2020 Jun 5;8:430. doi: 10.3389/fcell.2020.00430 (PMC7291606; doi:10.3389/fcell.2020.00430)

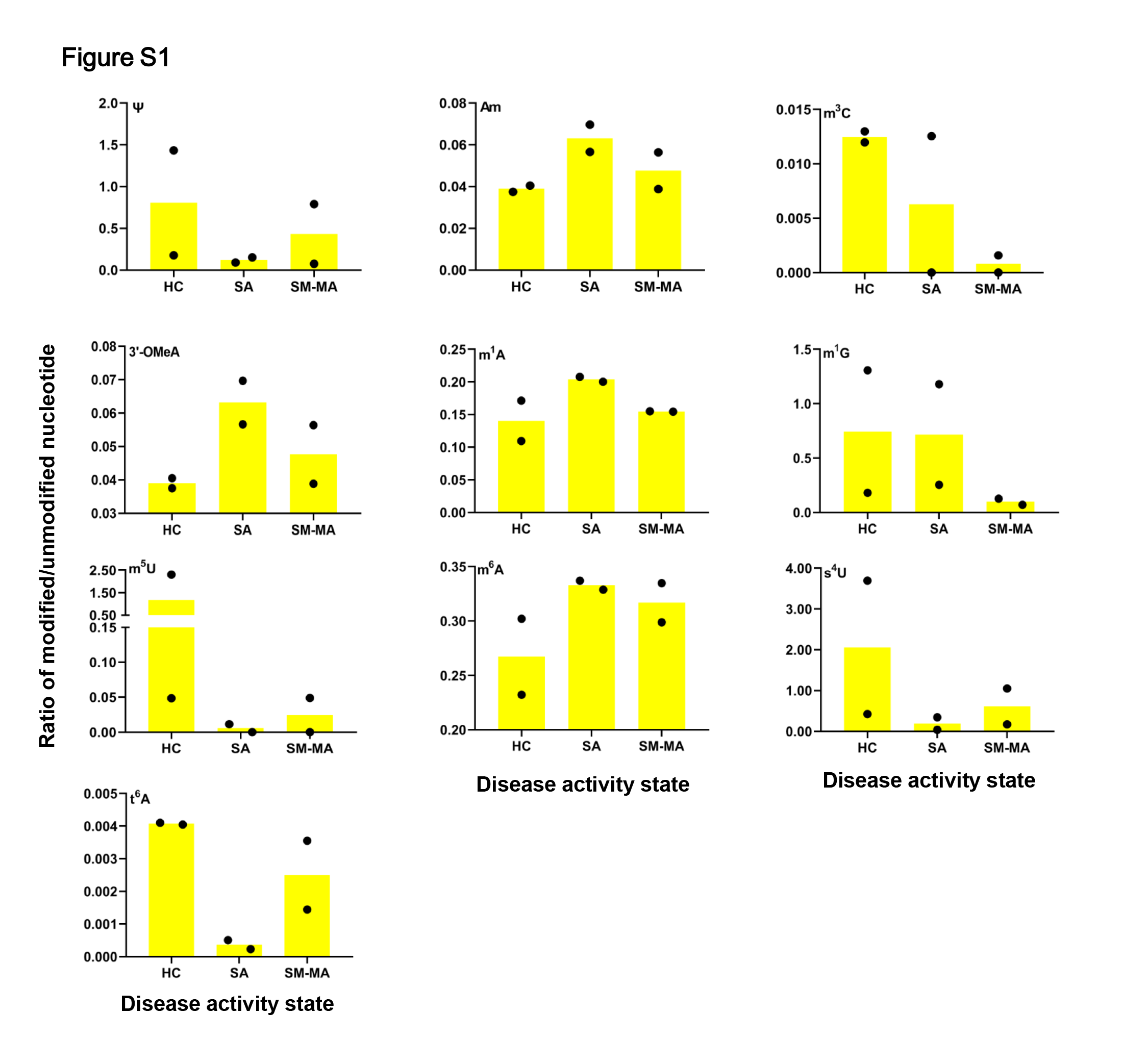

Supplement: FIGURE S1 — Global dynamics of mRNA modifications (calculated as ratio of modified/unmodified nucleotide). The maximal ratio of each modification was set to 1. Points represent individual biological replicates (n = 2) with bars showing the mean. [file Image_1.tif]

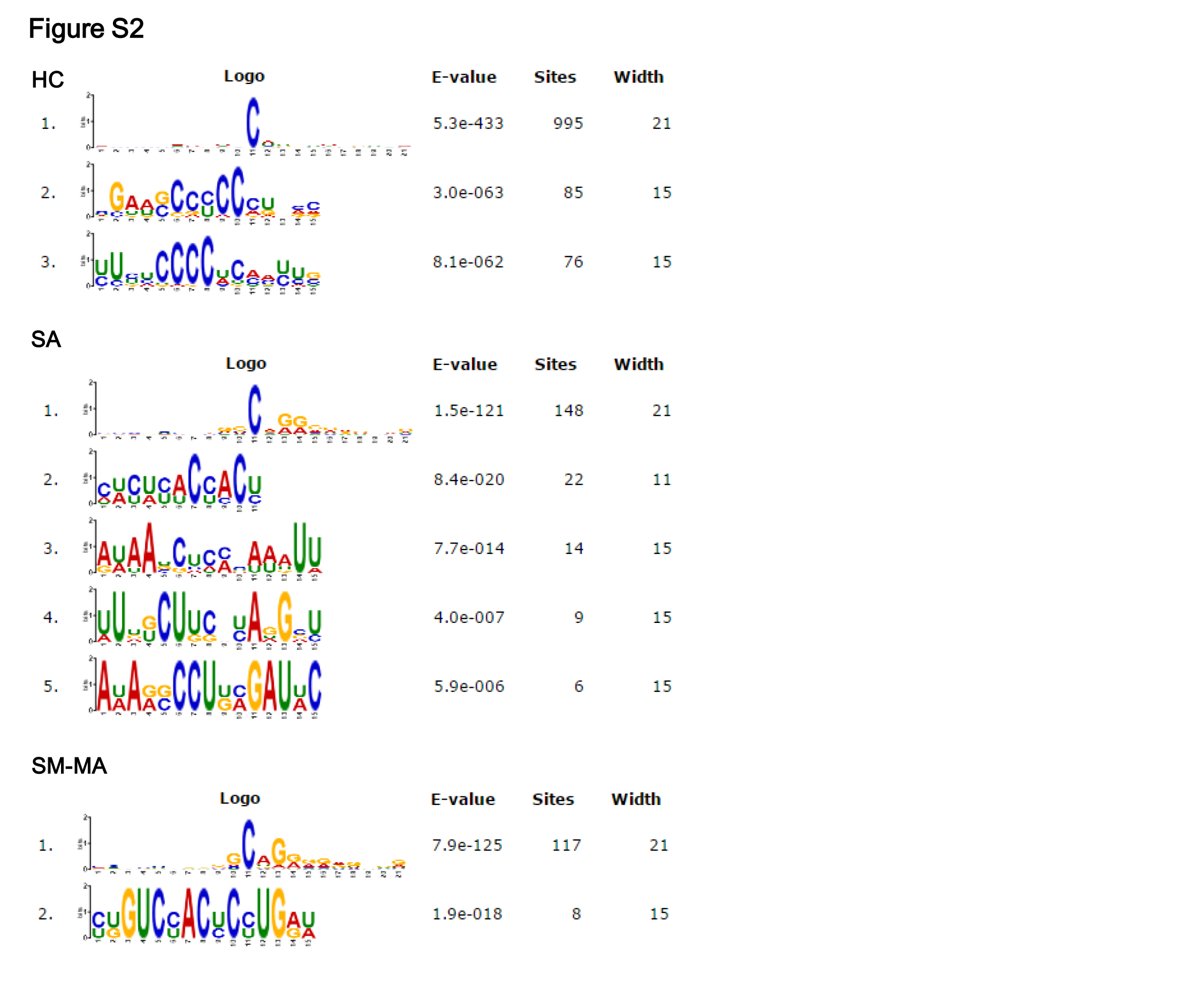

Supplement: FIGURE S2 — Top classic mode motifs enriched across the sequences proximal to mRNA m5C sites identified from healthy controls and SLE stable and SLE moderate/major active patients, respectively (only E-value < 0.05 is shown here). [file Image_2.tif]

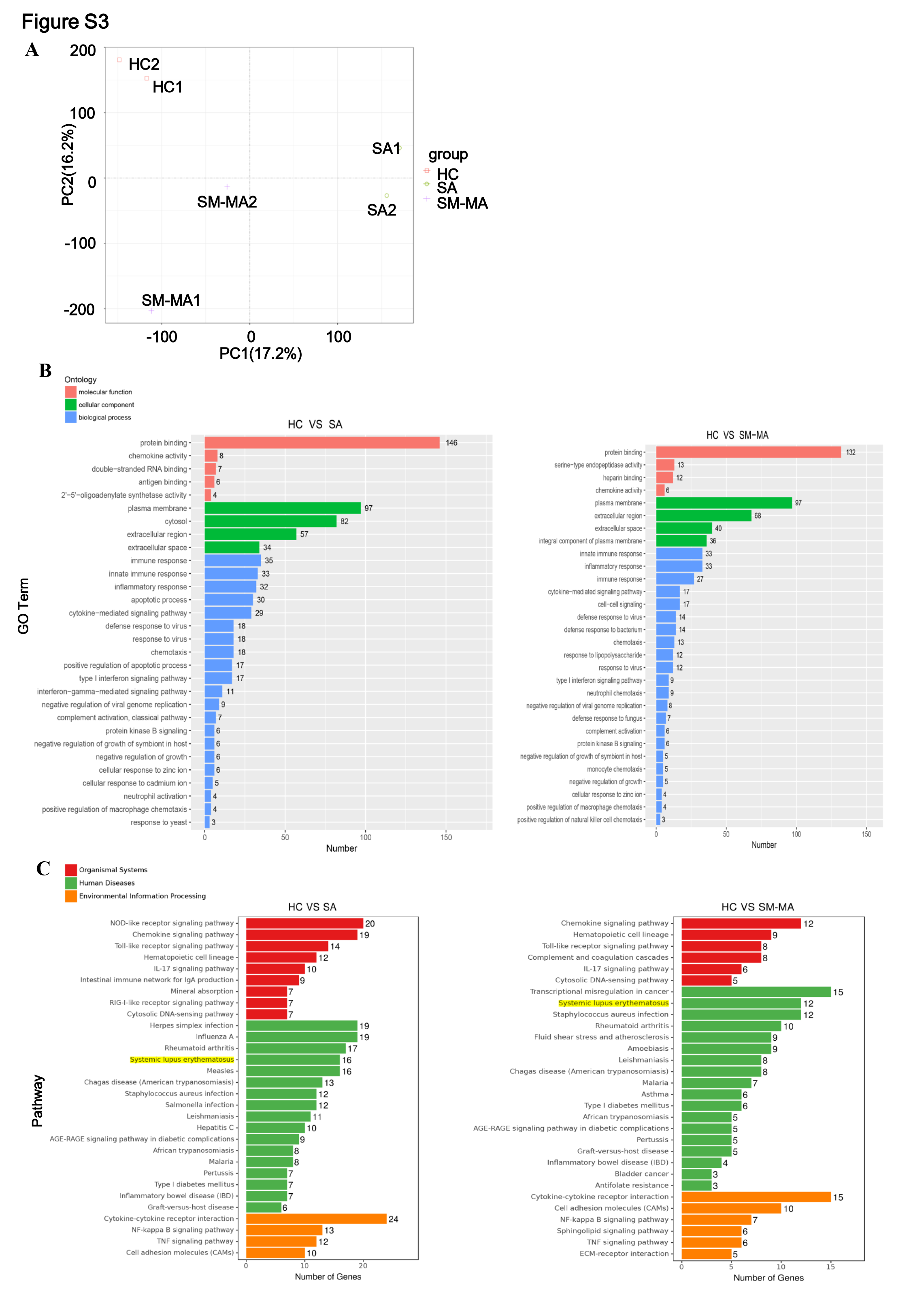

Supplement: FIGURE S3 — Pathway analysis of differentially expressed mRNAs in the CD4+ T cells from healthy controls (HCs) and systemic erythematosus lupus patients (SLE). (A) Principal component analysis (PCA) of the mRNA expression profiles in CD4+ T cells from HCs and SLE patients. This is another way to visualize sample-to-sample distances. In this ordination method, the data points are projected onto the 2D plane such that they spread out in the two directions that explain most of the differences. The x-axis separates the data points the most. The values of the samples in this direction are referred to as PC1. The y-axis (it must be orthogonal to the first direction) separates the data points the second most. The values of the samples in this direction are referred to as PC2. The percent of the total variance that is contained in the direction is shown on the axis label. Note that these percentages do not add to 100%, because there are more dimensions that contain the remaining variance (although each of these remaining dimensions will explain less than the two that we see). This analysis was performed using R language. (B) Gene ontology analysis of differentially expressed mRNAs. Red indicates molecular function (MF), green indicates cellular component (CC), and blue indicates biological process (BP). (C) Pathway analysis of differentially expressed mRNAs. Red indicates organismal systems, green indicates human diseases, and blue indicates environmental information processing. [file Image_3.tif]
